# Eco-Friendly Synchronous Spectrofluorimetric Determination of Imipenem, Cilastatin, and Relebactam; Application to Market Formulations and Biological Fluids; Greenness Assessment

**DOI:** 10.1007/s10895-024-04006-y

**Published:** 2024-11-15

**Authors:** Hesham Salem, Mahmoud A. Abdelmajed, Nadeen Emad, Belal M. Abdelghany, Anas Mahmoud, Amir Ata, Mahmoud Abdelgaleel

**Affiliations:** Pharmaceutical Chemistry Department, Faculty of Pharmacy, Deraya University, New Minia, Egypt

**Keywords:** Relebactam, Cilastatin, Imipenem, Synchronous fluorescence, Fourier self-deconvlution.

## Abstract

The proposed study introduces a rapid, sensitive, and simple synchronous spectrofluorimetric technique for simultaneous quantification of relebactam, cilastatin, and imipenem in marketed pharmaceutical forms and biological fluids. Using synchronous fluorescence spectroscopy at Δ λ = 110 nm, cilastatin was detected at 360 nm. Fourier Self-Deconvolution was subsequently applied to the spectrum to estimate relebactam and imipenem at 430 nm and 470 nm, respectively after detection of cilastatin at 360 nm ensuring no cross-interference. The pH was adjusted to 8.0 using 2.0 mL of alkaline borate buffer. This approach allowed for the precise quantification of relebactam, cilastatin, and imipenem through ranges of 50–400 ng mL^− 1^, 20–500 ng mL^− 1^, and 50–500 ng mL^− 1^ respectively. The lower detection and quantitation limits were 9.9 and 29.7 ng mL^− 1^ for REL, 4.5 and 13.6 ng mL^− 1^ for CIL and 5.5 and 16.5 ng mL^− 1^ for IMP. The proposed method was successfully applied for the determination of studied drugs in their pharmaceutical formulations with a high degree of accuracy and without interference from common excipients. This approach allowed for the precise quantification of relebactam, cilastatin, and imipenem through ranges of 50–400 ng mL^− 1^, 20–500 ng mL^− 1^, and 50–500 ng mL^− 1^, respectively. The proposed method was rigorously validated according to ICH guidelines. Furthermore, the method’s environmental impact was assessed using Eco-scale and Green Analytical Procedure Index (GAPI) techniques.

## Introduction

**Fig. 1:**
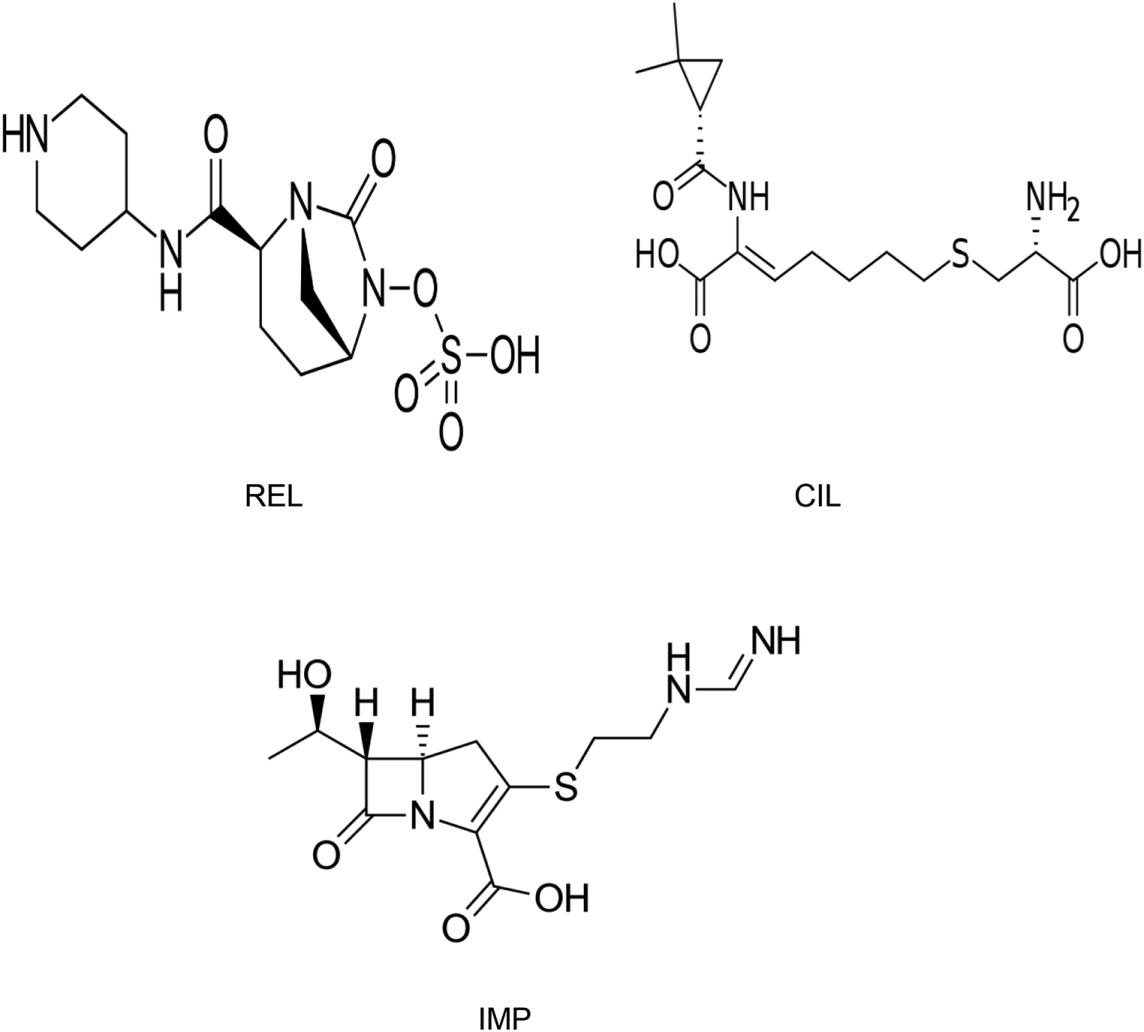
Chemical structures of REL, CIL and IMP

Imipenem (IMP) is (5R,6 S)-3-({2-[(E) (aminomethylidene) amino] ethyl} sulfanyl)-6-[(1R)-1-hydroxyethyl]-7-oxo-1-azabicyclo [3.2.0] hept-2-ene-2-carboxylic acid (Fig. 1), is a crystalline derivative of the novel carbapenem antibiotic thienamycin. It is renowned for its potent antibacterial activity against a wide spectrum of Gram-positive and Gram-negative bacteria [1–3]. Due to its rapid metabolism in the kidney [4], IMP is administered in combination with cilastatin (CIL) to inhibit renal dehydropeptidase and prevent the degradation of imipenem, thereby enhancing its efficacy and prolonging its antibacterial action.

CIL, disodium (2Z)-7-{[(2R)-2-amino-2-carboxyethyl]sulfanyl}-2-{[(1 S)-2,2-dimethylcyclopropyl] formamido} hept-2-enoic acid (Fig. 1), is a renal dehydro-peptidase inhibitor used to prolong the half-life of IMP and retard its inactivation [5]. Relebactam (REL) is [(2 S,5R)-7-oxo-2-(piperidin-4-ylcarbamoyl)-1,6-diazabicyclo [3.2.1 ]octan-6-yl] hydrogen sulfate (Fig. 1) is a β-lactamase inhibitor with the ability to inhibit a broad spectrum of β-lactamases such as class A and class C β‐lactamases, including carbapenemases. A new β-lactam–β-lactamase inhibitor combination called Imipenem-cilastatin-sulbactam (IMI-REL) was recently licensed to treat difficult intra-abdominal infections and complex urinary tract infections. Relebactam combined with imipenem makes it effective against a variety of imipenem-resistant bacteria, such as Pseudomonas aeruginosa and Enterobacteriaceae [6]. Several methods were reported for the analysis of IMP as a single drug or in the presence of other antibiotics or co-administered drugs. These methods include chromatographic methods [7–21], spectroscopic methods [22–25], or electroanalytical methods [26–28]. The combination of IMP and CIL was also quantified by several analytical methods such as chromatographic methods [29–31] and spectrophotometric methods [32–35]. Currently, only one HPLC chromatographic method [36] has been published for simultaneous determination of REl, CIL, and IMP. This means that new analytical methods were required for simultaneous analysis of those medications. The current work aims to establish the first spectroscopic methodology that can estimate these drugs in both dosage forms and biological fluids incorporating the principles of green chemistry. Fluorescence techniques have many advantages, such as speed and simplicity with low cost, as well as high selectivity, specificity, and sensitivity. In addition, fluorescence is far more sensitive than absorption spectroscopy, where it depends on measurement of the beam intensity directly with low background rather than comparison to a reference beam, and more preferable than chromatography as a result of the lack of mobile phase preparation and conditioning stages necessary for chromatographic methods, which boosts the tool’s productivity. Fluorescence techniques can be applied for quantification and detection of drugs in pharmaceutical dosage forms, biological fluids.

As part of a green chemistry strategy, Environmental Protection Agency staff have begun establishing safety and health protocols in an effort to minimize or restrict the manufacture of hazardous and toxic chemicals. Molecularly, it avoids contamination and reduces the negative effects of chemical goods on human health and the environment by reducing environmental pollution. By managing waste product quantities and waste treatment performance, the green approach might be accomplished by decreasing pollution, using less hazardous solvents and removing solvents from their source by regulating waste product volumes and waste treatment effectiveness. The Eco-scale is successfully employed to assess the impact of reagents, instruments, and waste. In contrast, the GAPI method offers a more detailed quantitative evaluation of all procedures of the approach [37, 38]. Techniques of fluorimetry are highly regarded for detecting a variety of components, though they can struggle with selectivity due to overlapping spectra in multi-component mixtures. By utilizing synchronous and deconvolution detection modes, these spectral overlap issues were mitigated, resulting in reduced spectral bands and improved band resolution [39–44].

## Experimental

### Apparatus

An FP-6200 Spectrofluorimeter named Jasco conducted with a 150-watt Xenon lamp and quartz cell with 1 cm was employed to establish the methodology’s analysis. Other equipment employed included a Precisa 125 A analytical balance (Switzerland) and pH meter (Jenway 3510, England), and a Scilogex RE 100-pro rotary evaporator.

### Chemicals, Reagents and Dosage Form

Authentic powders of CIL (99.56%), REL (99.31%) and IMP (99.04%) were kindly provided by (EIPICO Company, Egypt). Fresh plasma specimens were generously gifted by Hospital of Minia University (Minia, Egypt) and stored at -29 °C until they were analyzed. During the investigation, new distilled water was produced and all compounds utilized were of analytical quality, along with solvents of HPLC grade. Ethanol, methanol, acetone, and acetonitrile were sourced from Sigma-Aldrich, Germany. Hydrochloric acid, sodium hydroxide, boric acid, potassium phosphate monobasic, potassium chloride, and biphthalate (all these chemicals were adjusted as 0.2 M solutions) and purchased from El Nasr Company, Cairo, Egypt [45].

**Spectopenem**^®^ Vial (500 CIL and 500 IMP/ Vial), manufactured by EIPICO (Egypt), was bought from the local market. **Recarbrio**^®^ Vial (500 CIL, 500 IMP, and 250 REL/ Vial), manufactured by Merck & Co., Inc.

### Preparation of Solutions

One mg mL^-1^ concentration of stock solutions of REL, CIL, and IMP were adjusted by transferring 100 mg of each powder into 100-mL volumetric flasks, then completed to mark with distilled water. Then, further dilution with the same diluent was implemented to prepare a working standard solution with a concentration of 10 µg mL^-1^.

### Fluorescence Procedure

Different volumes from the working solution (10 µg mL^-1^) for REL, CIL, and IMP corresponding to (50–400, 20–500, and 50–500 ng mL^-1^) respectively, were poured into a series of 10-mL volumetric flasks using a micropipette. To each flask, 2 mL of alkaline borate buffer (pH 8) was added. The volume was then adjusted to the mark with the experimental diluent. To get the synchronous fluorescence spectra, an unchanged wavelength difference (Δλ) related to the two monochromators were (190, 110, and 190 nm) for REL, CIL, and IMP, subsequently. Each synchronous fluorescence spectrum was subjected to Fourier Self-Deconvolution using a full width at half maximum (FWHM) of 110 nm. For the three drugs respectively, REL, CIL, and IMP Peak amplitudes for the deconvoluted synchronous spectra were measured at 430, 360, and 470 nm. The calibration curves were generated by plotting the estimated values versus each medication concentration. A blank experiment was conducted in parallel procedures.

### Analysis of Laboratory-Prepared Mixtures

Various aliquots of REL, CIL, and IMP were taken from their corresponding working standard solutions into 10-mL volumetric flasks for this investigation. These aliquots were subsequently diluted with the experimental diluent to reach their final concentrations. Consequently, complementary ratios of each medication were created in mixes that remained within their linear range. The REL, CIL, and IMP concentration levels were estimated in each laboratory-prepared combination utilizing similar methods, as previous outlined.

### Optimization of Conditions

Throughout all trials, the concentrations of REL, CIL, and IMP were maintained at 200, 350, and 350 ng mL^-1^, correspondingly.

Utilizing a range of Δλ (50–250 nm), the impact of Δλ was examined. The capacity to resolve the overlapping spectra of the three medications and the values of intensity of the fluorescence were then compared.

An analysis was conducted to assess the effects of several diluting solvents on fluorescence intensity, such as distilled water, ethanol, methanol, acetone, and acetonitrile.

The effects of pH and buffering conditions were evaluated by comparing fluorescence intensity levels using buffers with different pH values.

To examine how buffer volume affects the study, the alkaline borate buffer was employed in varying amounts at pH 8, and the values of intensity of the fluorescence were recorded.

The separation efficacy of the three cited drugs was evaluated through the estimation of FWHM parameter. FWHM values in a range of 20 to 140 nm were examined. The potential to distinguish between the overlapping spectrum and values of intensity of the fluorescence were compared for each medication.

### Analysis of Pharmaceutical Formulation

Five Recarbrio^®^ vials (400 mg IMP, 400 mg CIL, and 200 mg REL / vial) were evacuated and finely powdered. An appropriate amount of the powder equal to 10 mg (for both IMP and CIL) and 5 mg of REL was accurately weighed and placed into a 100-mL volumetric flask. Distilled water was added until the volume reached approximately 70 mL, and the mixture was shaken vigorously for 15 min and completed to mark with the same diluent. The solution was then filtered and further dilution was implemented to achieve the desired concentration.

Fife Spectopenem^®^ vials (500 mg IMP and 500 mg CIL / vial) were evacuated and finely powdered. An exact quantity of powder, corresponding to 10 mg each of IMP and CIL, was precisely weighed and added to a 100-mL volumetric flask. Distilled water was then added to bring the volume to approximately 70 mL. After vigorous shaking for 15 min, additional distilled water was introduced to reach the necessary final volume. The resulting solution was filtered and diluted further with the same diluent to achieve the desired concentration.

The reported methodology involves using a reverse-phase high-performance liquid chromatography (RP-HPLC) system with a C18 column. Acetonitrile acts as the organic modifier, while the aqueous phase is composed of 0.03 M dipotassium hydrogen phosphate, with the pH adjusted to 3.2 using 0.1% (v/v) ortho-phosphoric acid. The mobile phase is delivered at a flow rate of 1.0 mL per minute. UV detection occurs at 265 nm. The observed retention times are 3.107 min for IMP, 3.885 min for CIL, and 10.516 min for REL [36].

### Application to Spiked Human Plasma

Separate 10 mL centrifuge tubes, 1 mL of the human plasma and 3 mL of acetonitrile was added, respectively, each holding 1 mL of drug-free plasma, were pipetted with varying quantities of REL, CIL, and IMP standard solutions (10 µg mL^− 1^). To precipitate proteins, 3 mL of methanol was added and the contents were thoroughly mixed using a vortex shaker. Following centrifugation at 4000 rpm for 30 min, A vacuum rotary evaporator was used to evaporate the protein-free supernatants until they were completely dry. Following that, they were put back into 10 mL volumetric flasks and diluted to a final amount of 10 mL using distilled water and 2 mL of alkaline borate buffer (pH 8). This process was repeated for each drug within their respective working concentration ranges. The concentrations of REL, CIL, and IMP were quantified using suitable regression equations.

## Results and Discussion

### Method Development and Optimization

In this research, REL, CIL, and IMP concentrations in marketed products and biological fluids were estimated utilizing a sensitive deconvoluted synchronous spectrofluorimetric method. Compared to conventional intrinsic fluorescence methods, this method narrows bands and limits spectrum range, simplifying emission spectra and enabling simultaneous analysis. Synchronous spectrofluorimetry produces sharp, narrow peaks because of its increased selectivity, which removes the requirement for multi-component sample separation beforehand. Resolution was effectively enhanced by deconvolution through compressing spectral bandwidths, distinguishing each component clearly. Our approach allows for concurrent quantification of REL, CIL, and IMP in both bulk and plasma samples using synchronous spectrofluorimetry combined with deconvolution.

**Fig. 2:**
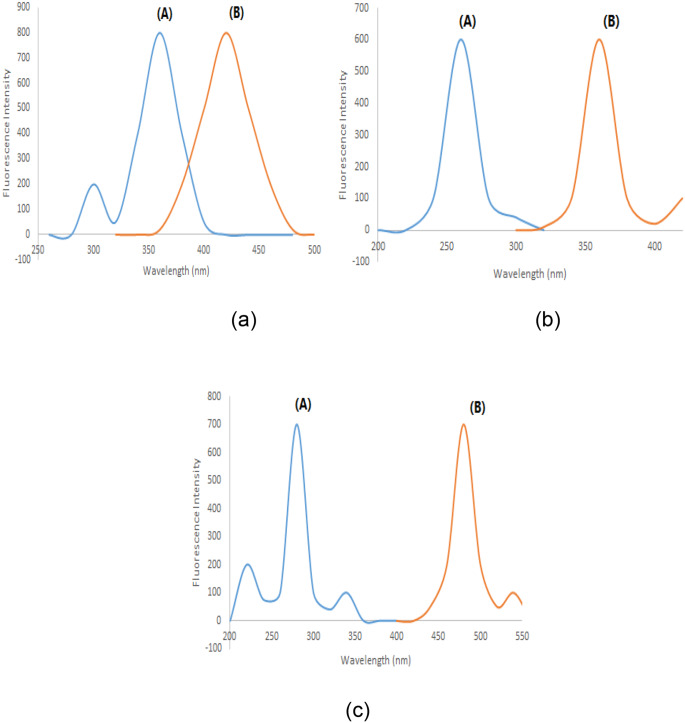
(A) Excitation and (B) emission spectra of REL 200 ng mL^− 1^ (**a**), CIL 350 ng mL^− 1^ (**b**) and IMP 350 ng mL^− 1^ (**c**), in distilled water

**Fig. 3:**
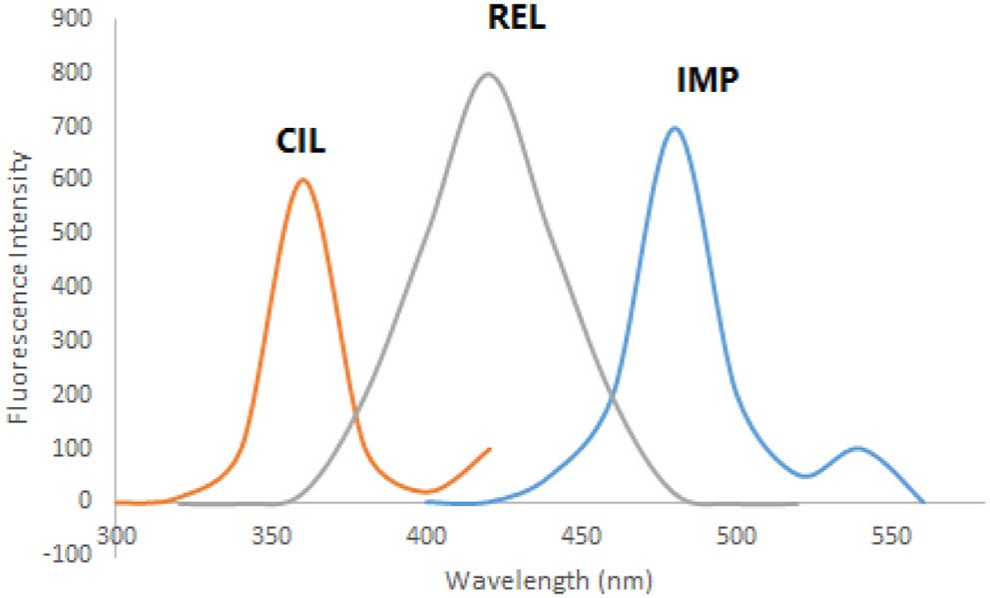
Emission spectra of 250 ng mL^− 1^ REL, 50 ngmL^− 1^ CIL and 450 ng mL^− 1^ IPM in distilled water upon excitation

**Fig. 4:**
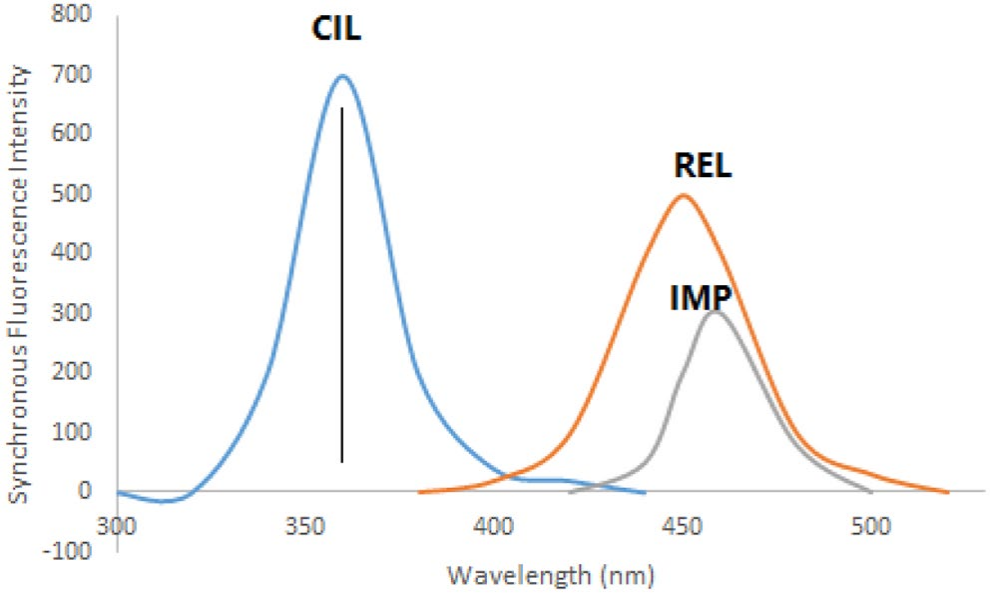
Synchronous fluorescence spectra of 200 ng mL^− 1^REL, 350 ng mL^− 1^ CIL and 350 ng mL^− 1^ IMP using Δ λ = 110 nm

**Fig. 5:**
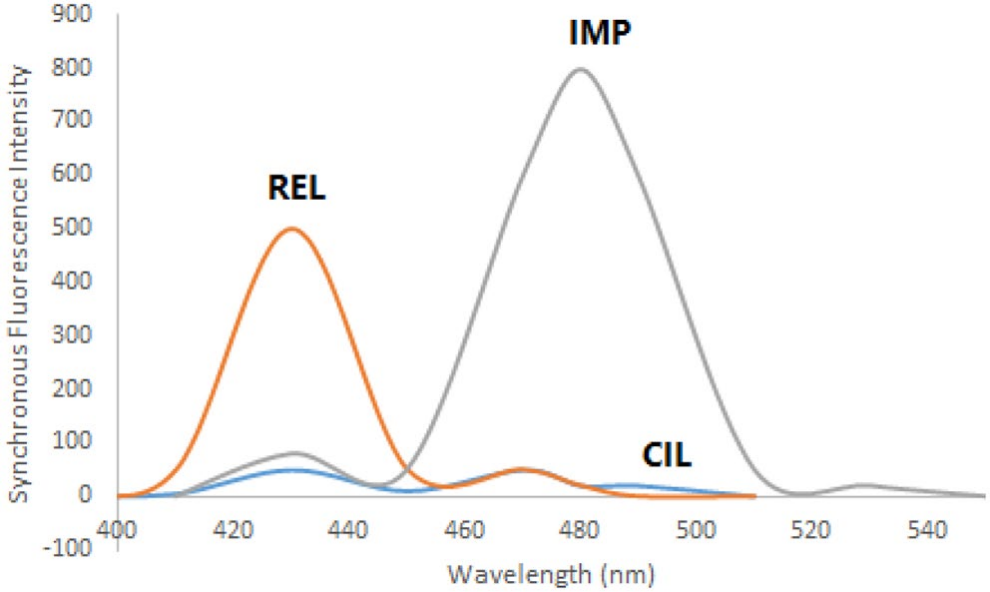
Synchronous fluorescence spectra of 200 ng mL^− 1^ REL, 350 ng mL^− 1^ CIL and 350 ng mL^− 1^ IMP using Δ λ = 190 nm

**Fig. 6:**
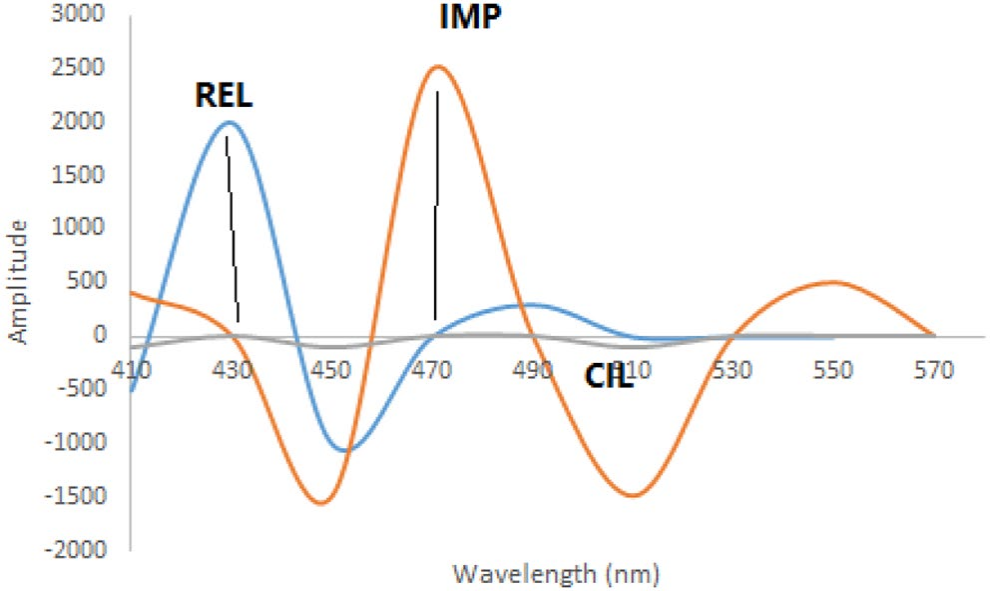
Deconvoluted synchronous fluorescence spectra of 200 ng mL^− 1^ REL, 350 ng mL^− 1^ CIL and 350 ng mL^− 1^ IMP using synchronous Δ λ = 190 nm

REL, CIL, and IMP exhibit intrinsic fluorescence characteristics. REL displays excitation peaks at 300 and 360 nm with an emission peak at 420 nm (Fig. 2a). CIL exhibits an excitation peak at 260 nm and an emission peak at 360 nm (Fig. 2b), while IMP features excitation peaks at 220, 280, and 340 nm with an emission peak at 480 nm (Fig. 2c). The overlapping emission spectra of these drugs (as illustrated in Fig. 3) present challenges for conventional fluorescence methods. However, synchronous fluorescence measurement at Δλ = 110 nm allows distinct estimation of CIL at 360 nm free from the other medications interfering (as shown in Fig. 4). To address overlap between REL and IMP, different wavelength settings, such as Δλ = 190 nm (Fig. 5), were explored, followed by Fourier Self Deconvolution utilizing FWHM of 110 nm. Because of the efficient overlap resolution provided by this technique, REL at 430 nm and IMP at 470 nm may be measured without external interference as shown in (Fig. 6).

The study thoroughly investigated and optimized all conditions affecting the synchronous fluorescence intensity of REL, CIL, and IMP. All the experimental factors were adjusted independently while freezing the others. Like, buffer condition, Δλ for synchronous scanning, diluting solvent, and FWHM.

To effectively separate spectra in synchronous mode, a value of 110 nm was found to be appropriate. This allowed for the partial resolution of overlapping spectra and the particular measurement of CIL at 360 nm without interference from other medicines. The Fourier Self Deconvolution approach with FWHM = 110 nm was shown to be successful in further resolving the overlap between REL and IMP when Δλ was increased to 190 nm.

**Fig. 7:**
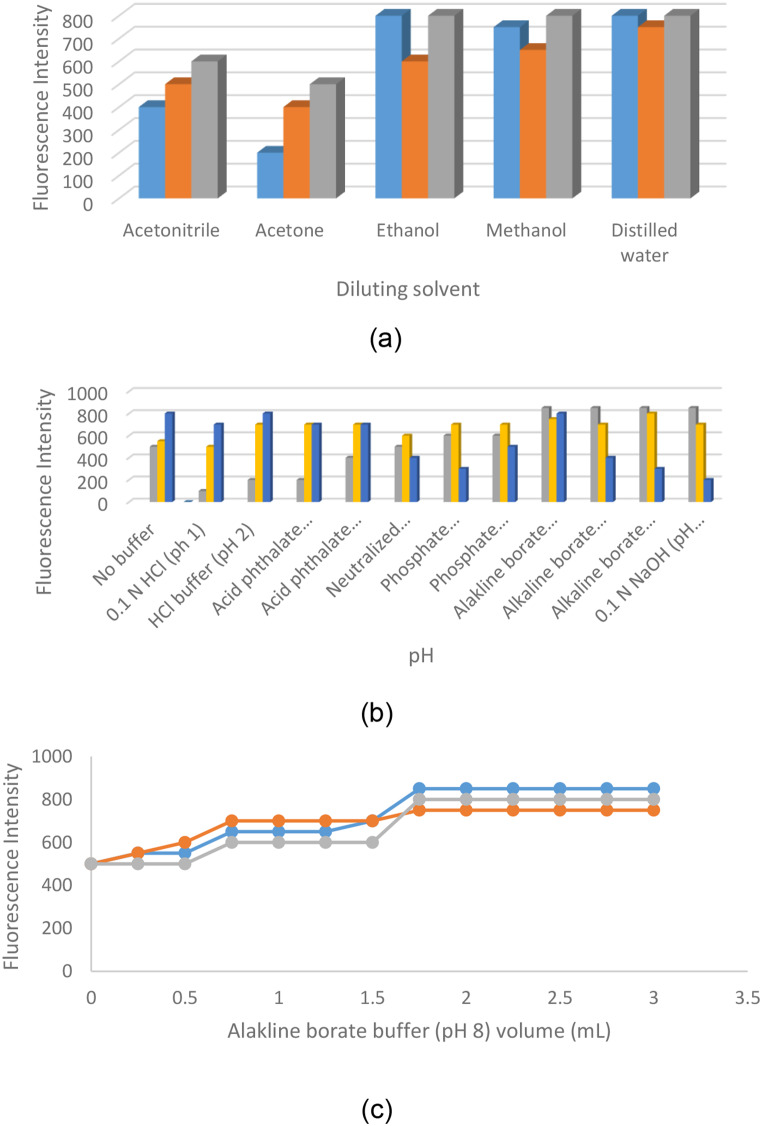
Effect of diluting solvent (**a**), buffer type (**b**) and buffer volume (**c**) for REL (200 ng mL^− 1^), CIL (350 ng mL^− 1^) and IMP (350 ng mL-1)

Various diluting solvents were tested, with ethanol, methanol, and distilled water proving most effective in enhancing fluorescence intensity. Distilled water was selected for its environmentally friendly properties, diluents like acetonitrile and acetone exhibited lower fluorescence sensitivity (all shown in Fig. 7).

The media pH was tested, revealing that the optimum pH resulting the highest intensity of fluorescence was 8. Additionally, buffer volume was evaluated, demonstrating that 2 mL was sufficient for the best proposed analysis, (as illuminated in Fig. 7)

### Method Validation

The suggested fluorimetric methodology was rigorously validated according to (ICH) [46] guidelines. The process included assessments of accuracy, precision, specificity, linearity, and robustness. The results of these evaluations demonstrated that the method met al.l the required criteria, confirming its reliability and robustness for the intended analytical applications.

### Linearity and Range

Under the specified experimental conditions, calibration graphs for REL and IMP were generated by plotting each medication concentration versus its synchronous fluorescence intensity, which ranged from 50 to 400 ng mL^-1^ for REL and 50–500 ng mL^-1^ for IMP. For CIL, a calibration graph was created by plotting fluorescence intensities against concentrations spanning from 20 to 500 ng mL^-1^. Analytical values related to the regression equation, as detailed in (Table 1), demonstrated high coefficients of determination, confirming the excellent linearity of the calibration graphs.

**Table 1:** Analytical factors related to the regression equation and validation parameters of the studied approach

Parameters	REL	CIL	IMP
Wavelength (nm)	430	360	470
Linearity range (ng mL^− 1^)	50–400	20–500	50–500
Slope ± SD	5.40 ± 0.06	7.78 ± 0.03	7.88 ± 0.04
Intercept ± SD	-70.00 ± 16.07	-36.48 ± 10.56	-152.19 ± 13.04
LOD (ng mL^− 1^)	9.91	4.48	5.51
LOQ (ng mL^− 1^)	29.75	13.58	16.55
Coefficient determination (r^2^)	0.9996	0.9997	0.9999
Accuracy (%R)^a^	100.67	100.94	99.95
**Precision (%RSD)** ^**b**^			
- intraday precision (repeatability)	1.25	1.04	1.861
- Inter day precision	1.57	1.38	1.11
**Robustness (%R ± %RSD)**			
Δλ (± 2 nm)	100.43 ± 0.89	98.94 ± 0.85	101.01 ± 1.03
pH (± 0.1)	99.43 ± 0.95	100.43 ± 0.97	100.47 ± 1.05
Borate buffer volume (± 0.1 mL)	100.42 ± 0.89	99.21 ± 1.04	99.31 ± 1.63

^a^ Average of determinations (3 concentrations repeated 3 times)

^b^ %RSD of 9 determinations (3 concentrations repeated 3 times)

The limits of detection (LOD) and quantification (LOQ) were determined and the results are presented in (Table 1). These findings highlight the sensitivity of this strategy.

### Accuracy and Precision

In order to achieve repeatability and intermediate precision, the recommended strategy was utilized to estimate different levels of concentration 3 times (50, 100, and 200 ng mL^− 1^ for REL, 50, 150, and 350 ng mL^− 1^ for CIL, and 50, 250, and 350 ng mL^− 1^ for IMP). These levels were estimated within a single day and through 3 successive days. Excellent % R and small values of RSD respectively demonstrate how the suggested approach’s high level of accuracy and precision. All data was inserted in (Table 1).

### Specificity

Preparing combinations in the lab with varying proportions of the three medications was crucial. Utilizing the methods outlined in Sect. 2.4.1, these mixes were examined. The results, which are summarized in (Table 2), were found to be satisfactory and acceptable. The technique of Standard addition was employed on the previously determined specimens to examine the effect of excipients on the drug quantification, as shown in (Table 3). The successful application of this technique confirmed the method’s selectivity and its ability to prevent interference from excipients.

**Table 2:** Analysis of laboratory prepared mixture of REL, CIL and IMP by the proposed spectrofluorimetric method

Laboratory prepared mixture (ng mL^− 1^)			% Recovery		
REL	CIL	IMP	REL	CIL	IMP
100	100	100	100.42	98.49	99.39
100	200	200	99.39	100.49	100.49
200	100	100	99.30	100.59	100.48
100	200	100	100.29	100.19	100.69
200	200	100	100.59	100.94	100.68
100	300	300	100.49	100.04	99.39
*Mean ± SD			100.08 ± 0.58	100.12 ± 0.86	100.19 ± 0.62

*Average of 3 determinations

**Table 3:** Application of standard addition technique using the proposed methodology

Dosage Forms	CIL		IMP		REL	
**Spectopenem**^®^ Vial	Pure added (ng mL^− 1^)	% R	Pure added (ng mL^− 1^)	% R	-	-
500 CIL	50	100.65	75	99.30		
500 IMP	100	100.04	150	100.83		
	150	100.86	225	100.11		
Mean		100.52		100.08		
%RSD		0.42		0.76		
**Recarbrio** ^®^ **Vial**	Pure added (ng mL^− 1^)	% R	Pure added (ng mL^− 1^)	% R	Pure added (ng mL^− 1^)	% R
500 CIL	100	100.70	200	100.69	50	99.49
500 IMP	200	100.83	300	99.01	100	100.52
250 REL	300	100.99	400	100.50	150	101.01
*Mean		100.84		100.07		100.34
%RSD		0.14		0.92		0.77

*Average of three determinations

### Robustness

This validation tool was evaluated by applying minor changes in one parameter like synchronous Δλ, pH, the volume of buffer, and deconvolution Δλ while freezing the other parameters. Values of % R near 100% and % RSD under 2% indicated that the method’s synchronous deconvolution process remained largely unaffected by the changes in these parameters.

### Application to Pharmaceutical Formulation

The outcomes obtained from the technique of standard addition, as shown in (Table 3), conclusively revealed that the presence of excipients or additives did not influence the accuracy of the analysis. In the proposed method, REL, CIL, and IMP were analyzed in two different formulations: firstly, as a binary mixture of CIL and IMP in Spectopenem^®^ Vials, and secondly, as a tertiary mixture of REL, CIL, and IMP in Recarbrio^®^ Vial. All values were gathered in (Table 4).

**Table 4:** Determination of REL, CIL and IMP in pharmaceutical dosage forms by the proposed spectrofluorimetric method

Spectopenem^®^ Vial				Recarbrio^®^ Vial							
CIL		IMP		REL		CIL			IMP		
Conc. ng mL^− 1^	% R	Conc. ng mL^− 1^	% R	Conc. ng mL^− 1^	% R	Conc. ng mL^− 1^		% R	Conc. ng mL^− 1^		% R
50	100.54	50	98.59	50	99.43	100		99.03	100		99.14
100	100.50	100	99.58	100	100.01	200		101.04	200		100.27
150	99.03	150	100.95	150	99.39	300		100.48	300		100.59
200	100.91	200	100.18	200	100.04	400		100.73	400		100.94
250	100.88	250	100.85	250	100.48	500		100.58	500		100.78
*Mean	100.37		100.03		99.87			100.37			100.34
**% SD**	0.77		0.98		0.46			0.78			0.72

*Average of 3 determinations

### Application to Spiked Human Plasma

The newly proposed spectrofluorimetric approach demonstrated its capability in accurately measuring REL, CIL, and IMP at various concentration levels in spiked plasma specimens, owing to its exceptional sensitivity. The concentrations of these drugs were determined using the corresponding regression equations provided in (Table 5).

**Table 5:** Determination of REl, CIL and IMP in spiked human plasma by the proposed spectrofluorimetric method

REL		CIL		IMP	
Taken (ng mL^− 1^)	% Recovery	Taken (ng mL^− 1^)	% Recovery	Taken (ng mL^− 1^)	% Recovery
50	93.29	100	94,01	50	94.19
50	94.03	150	95.29	75	95.67
100	93.93	200	96.29	75	96.30
100	92.98	200	95.33	100	96.20
150	94.96	250	94.98	100	94.05
*Mean	93.84		95.18		95.28
**SD**	0.77		0.82		1.09

*Average of 5 determinations

### Greenness Comparison

Using the student’s t-test and F-value at a 95% confidence level, the presented spectrofluorimetric methodology for analyzing the medications under study in their pharmaceutical dosage forms did not provide any statistically substantial alterations upon compared with the published one [36], as shown in (Table 6). This proves the accuracy and precision of the method presented.

**Table 6:** Statistical analysis and determination of REL, CIL and IMP in pharmaceutical preparations by the proposed spectrofluorimetric and reported methods

	Spectopenem^®^ Vial (CIL & IMP)		Recarbrio^®^ Vial (REL, CIL & IMP)	
	Proposed method	Reported method [31]	Proposed method	Reported method [36]
Number of measurements	5	5	5	5
Mean % Recovery	101.01	100.65	99.68	100.99
% RSD	0.79	1.13	1.57	1.01
Variance	0.69	0.79	1.70	0.90
Student’s, t-test	0.58		1.57	
F-value	2.04		2.36	

The values in parenthesis are tabulated values of t (2.306) and F (6.388) at (*P* = 0.05)

AES (analytical eco-scale) and GAPI (Green Analytical Procedure Index) were among the tools that evaluated the environmental impact of the presented approach [47–49]. AES evaluates penalty points based on instruments, type of reagents, and the yielded waste, providing a semi-quantitative assessment where deducting these points from 100 indicates the method’s environmental sustainability. (Table 7) shows that the proposed technique accrued 11 penalty points, resulting in a score of 89, indicating its superior environmental performance compared to traditional HPLC techniques. The method substitutes organic solvents with distilled water and avoids energy-intensive processes exceeding 0.1 kWh per sample. Utilizing a five pictogram, GAPI could assess the environmental impact of each step in the methodology process. Each pictogram assesses different steps on a scale from green (low environmental impact) to yellow (medium) to red (high). Additionally, a circle symbol indicates if quantification is included in the method. The GAPI pictograms for the proposed spectrofluorimetric technique in (Table 7) demonstrate nine green zones, highlighting their environmental suitability for pharmaceutical applications. Furthermore, the GAPI pentagram reveals only three red zones and more green zones compared to other methods, indicating the method’s superior environmental profile.

**Table 7:**
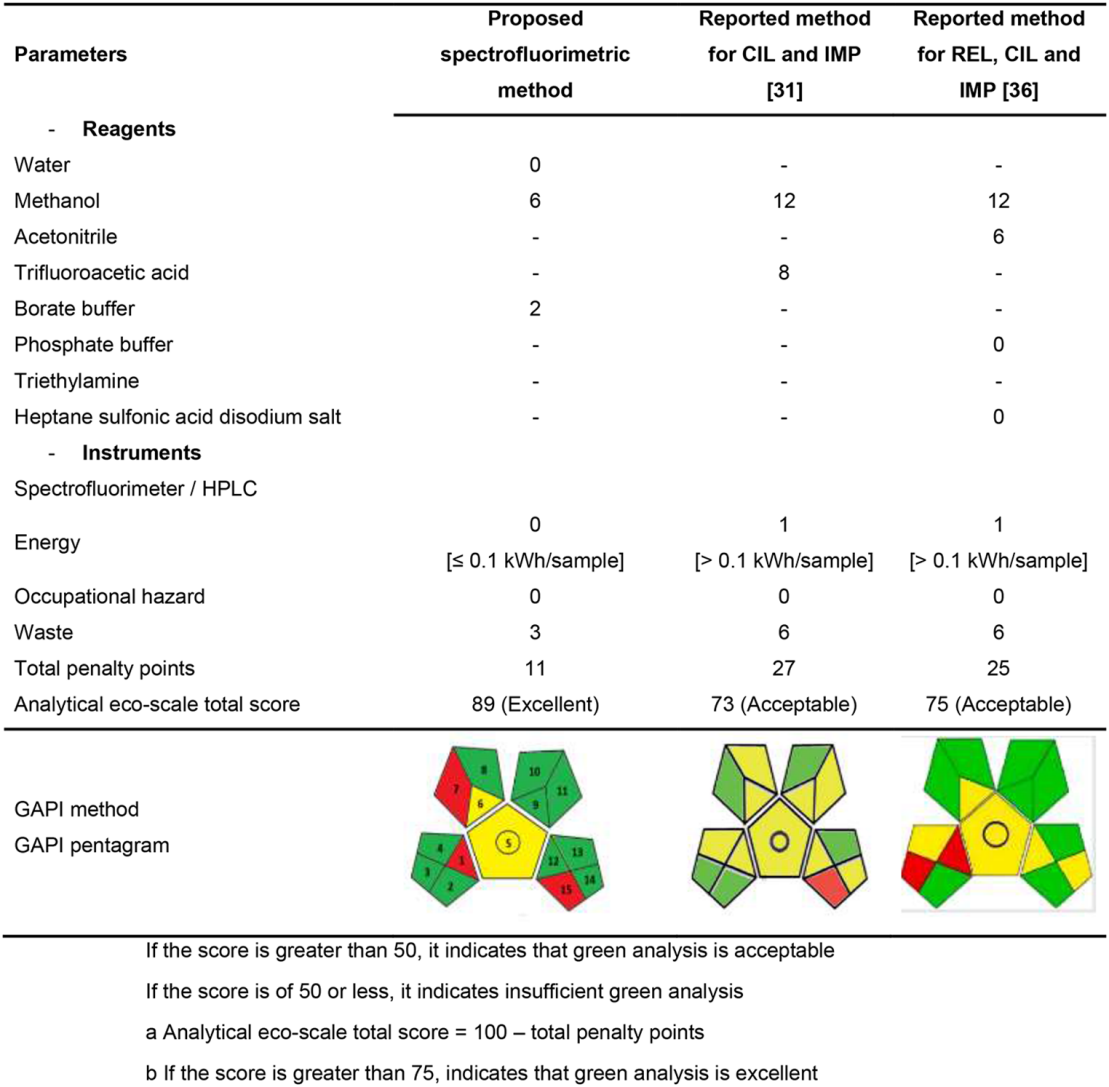
Greenness of the proposed spectrofluorometric and HPLC – reported techniques utilizing the eco-scale and GAPI tools

## Conclusion

In this study, the quantitative analysis of REL, CIL, and IMP in pharmaceutical formulations and spiked human plasma was conducted using synchronous fluorescence spectra. This method allows for the direct detection of CIL without interference from REL and IMP. Following this, spectral deconvolution facilitates precise quantification of REL and IMP individually. It is worth noting that current literature lacks environmental assessments for these drugs, especially in relation to plasma samples and the environmentally harmful solvents typically used in their extraction processes. Our novel approach addresses this deficiency by offering a more environmentally sustainable alternative.

In summary, the newly developed method presents several advantages over conventional techniques, including improved sensitivity, cost-effectiveness, time efficiency, and ecological benefits. This represents a significant advancement in pharmaceutical analysis, ensuring both accuracy and environmental conscientiousness.

## Data Availability

No datasets were generated or analysed during the current study.

## Declarations

### Application on Biological Fluids

The procedure was applied to spiked plasma only.
